# Sex-specific associations with cerebrospinal fluid biomarkers in dementia with Lewy bodies

**DOI:** 10.1186/s13195-020-00610-9

**Published:** 2020-04-17

**Authors:** M. van de Beek, R. Babapour Mofrad, I. van Steenoven, H. Vanderstichele, P. Scheltens, C. E. Teunissen, A. W. Lemstra, W. M. van der Flier

**Affiliations:** 1 Alzheimer Center Amsterdam & Department of Neurology, Neuroscience Campus Amsterdam Neuroscience, Vrije Universiteit Amsterdam, Amsterdam UMC, VU University Medical Center, Amsterdam, The Netherlands; 2 Neurochemistry Laboratory and Biobank, Department of Clinical Chemistry, Amsterdam Neuroscience, Amsterdam UMC, VU University Medical Center, Amsterdam, The Netherlands; 3 Biomarkable bvba, Ghent, Belgium; 4 Department of Epidemiology and Biostatistics, Vrije Universiteit Amsterdam, Amsterdam UMC, VU University Medical Center, Amsterdam, The Netherlands

**Keywords:** Dementia with Lewy bodies, Sex differences, Cerebrospinal fluid biomarkers

## Abstract

**Background:**

Dementia with Lewy bodies (DLB) is more prevalent in men than in women. In addition, post-mortem studies found sex differences in underlying pathology. It remains unclear whether these differences are also present antemortem in in vivo biomarkers, and whether sex differences translate to variability in clinical manifestation. The objective of this study was to evaluate sex differences in cerebrospinal fluid (CSF) biomarker concentrations (i.e., alpha-synuclein (α-syn), amyloid β1-42 (Aβ42), total tau (Tau), phosphorylated tau at threonine 181 (pTau)) and clinical characteristics in DLB.

**Methods:**

We included 223 DLB patients from the Amsterdam Dementia Cohort, of which 39 were women (17%, age 70 ± 6, MMSE 21 ± 6) and 184 men (83%, age 68 ± 7, MMSE 23 ± 4). Sex differences in CSF biomarker concentrations (i.e., α-syn, Aβ42, Tau, and pTau) were evaluated using age-corrected general linear models (GLM). In addition, we analyzed sex differences in core clinical features (i.e., visual hallucinations, parkinsonism, cognitive fluctuations, and REM sleep behavior disorder (RBD) and cognitive test scores using age- and education-adjusted GLM.

**Results:**

Women had lower CSF α-syn levels (F 1429 ± 164 vs M 1831 ± 60, *p* = 0.02) and CSF Aβ42 levels (F 712 ± 39 vs M 821 ± 18, *p* = 0.01) compared to men. There were no sex differences for (p) Tau concentrations (*p* > 0.05). Clinically, women were older, had a shorter duration of complaints (F 2 ± 1 vs M 4 ± 3, *p* < 0.001), more frequent hallucinations (58% vs 38%, *p* = 0.02), and scored lower on MMSE and a fluency task (MMSE, *p* = 0.02; animal fluency, *p* = 0.006). Men and women did not differ on fluctuations, RBD, parkinsonism, or other cognitive tests.

**Conclusions:**

Women had lower Aβ42 and α-syn levels than men, alongside a shorter duration of complaints. Moreover, at the time of diagnosis, women had lower cognitive test scores and more frequent hallucinations. Based on our findings, one could hypothesize that women have a more aggressive disease course in DLB compared to men. Future research should investigate whether women and men with DLB might benefit from sex-specific treatment strategies.

## Background

Dementia with Lewy bodies (DLB) is clinically characterized by cognitive decline, visual hallucinations, parkinsonism, cognitive fluctuations, and rapid eye movement (REM) sleep behavior disorder (RBD) [1]. Pathologically, DLB is characterized by the presence of cortical Lewy bodies, i.e., neuronal inclusions of alpha-synuclein proteins [2], frequently combined with Alzheimer’s disease (AD) pathology, i.e., amyloid plaques and neurofibrillary tangles (NFT) [3]. Most studies report a higher prevalence of DLB in men than in women [4–7], although a few report the opposite [8, 9]. In addition to the skewed distribution, there is evidence of differences in the underlying pathology between women and men with DLB. Post-mortem examinations showed that men were more likely to die from “pure” DLB pathology than women, and women were more likely to have mixed pathology (DLB + AD) [10, 11]. This is clinically relevant, as mixed pathology is linked to a more severe disease manifestation, with more cognitive disturbances, more frequent hallucinations, and shorter survival [12, 13]. Although post-mortem studies are important, a major drawback is that they reflect end-stage disease. In contrast, cerebrospinal fluid (CSF) biomarkers enable in vivo studies in ongoing pathological processes. Previous CSF biomarker studies in AD have shown sex differences in CSF (p) Tau concentrations, suggesting a higher antemortem neurofibrillary tangle (NFT) load in women with AD [14–16]. Literature regarding sex differences in CSF biomarkers in DLB, however, is still limited. One small study reported lower levels of CSF alpha-synuclein in DLB women compared to DLB men, AD, and controls [17]. In addition, apart from one study showing that visual hallucinations were more common in women [18], the relationship between clinical symptoms and sex in DLB has neither been studied extensively either. In order to provide biomarker-guided personalized medicine in the future, it is essential to further clarify sex differences in DLB biomarkers and symptomatology. Therefore, in the current study, we aimed to examine sex differences in CSF biomarkers and clinical symptomatology in a large well-defined cohort of DLB patients.

## Methods

### Participants

We included 223 DLB patients who visited the Alzheimer Center Amsterdam between 1999 and 2019 from the Amsterdam Dementia Cohort based on the availability of CSF [19]. All patients received standardized screening at baseline, which included a semi-structured medical history interview; physical, neurological, and neuropsychological examinations; magnetic resonance imaging (MRI); electroencephalography (EEG) or magnetoencephalography (MEG); and laboratory tests. Diagnoses were made in a multidisciplinary consensus meeting based on the results obtained from the standardized screening [19]. DLB patients were diagnosed clinically according to the current consensus criteria for probable DLB [1, 20]. FP-CIT single-photon emission computed tomography (DAT-SPECT) was available in 103 patients and was positive for DLB diagnosis in 92 patients. DLB was confirmed at autopsy in 3 patients, of which 2 did not have a DAT-SPECT scan. Written informed consent was obtained from all study participants for the use of clinical data and biomaterial for research purposes.

### Measures

#### Clinical and cognitive features

We rated the core clinical features at baseline from preformatted questionnaires or retrospectively from patients’ medical charts. Hallucinations were scored according to the caregiver-rated neuropsychiatric inventory (NPI) that was available in 222 patients [21]. Parkinsonism was assessed with a preformatted checklist for extrapyramidal signs during the neurological exam (i.e., tremor, bradykinesia, and/or rigidity), data available for 216 patients. The semi-structured patient history interview was reviewed for information on fluctuations and RBD. Fluctuations were scored as being present when the patient or caregiver reported clear changes in attention and cognition during the day or between days. Information on fluctuations was available for 173 patients. REM sleep behavior disorder (RBD) was rated positive when caregivers reported that patients “act out” their dreams during their sleep. Information on RBD was available of 156 patients. Duration of complaints was systematically assessed during the patient history interview and was defined as the moment when the patient first noticed their cognitive complaints. Depressive symptoms were assessed using the 15-item Geriatric Depression Scale, with higher scores indicating more depressive symptoms [22]. Mini Mental State Examination (MMSE) was used to assess global cognition. Memory was assessed using the immediate and delayed recall of the Dutch version of the verbal learning test (RAVLT) [23]. Attention and speed was assessed using Trail Making Test (TMT)-A. Executive functioning was assessed using the ratio of TMT-B/TMT-A [24]. We calculated inverse scores for time-dependent tests (i.e., TMT-A and TMT-B), such that lower scores represent worse performance. Missing values of the TMT-B were imputed using the group ratio of TMT-B/TMT-A. Neuropsychological test scores were available for 205 patients, and test results were *Z*-transformed based on cognitively normal subjects (*n* = 533, 60 ± 10 years, 54%F, MMSE = 29 ± 1).

#### Apolipoprotein E genotyping

The QIAamp DNA blood isolation kit from Qiagen (Venlo, The Netherlands) was used to isolate DNA from 10-ml vacutainer tubes containing EDTA. This was followed by genotype determination using the LightCycler ApoE Mutation Detection Kit (Roche Diagnostics, GmbH, Mannheim, Germany). Subjects with at least one APOEe4 allele were defined as APOEe4 carriers, whereas no e4 allele defined subjects as APOEe4 non-carriers.

#### CSF analysis

For CSF collection and processing, standardized protocols were followed [25, 26]. CSF total α-syn concentrations were determined using a sandwich ELISA assay (developed by ADx NeuroSciences, Ghent, Belgium and commercialized by Euroimmun AG, Lübeck, Germany) [27]. To avoid blood contamination, α-syn measures with high hemoglobuline (Hb) concentrations (> 10 ng/ml) or high erythrocyte count (> 500 ery/ml) were excluded. CSF total α-syn was available for 127 patients. CSF Aβ1-42, Tau, and pTau concentrations were measured using a sandwich ELISA (Innotest, Fujirebio, Gent, Belgium), or the Elecsys Aβ42, Tau, and pTau (181P) CSF assays run on the *cobas e*601 analyzer (Roche Diagnostics GmbH). Elecsys values of Aβ42, Tau, and pTau were converted to Innotest values using previously described formulas [28]. Drift corrected continuous Aβ42 concentrations were used in the data analyses, and to define amyloid positivity, Aβ42 concentrations were dichotomized using a cutoff < 813 pg/ml [29]. CSF Aβ1-42, Tau, and pTau were available for all patients.

### Statistical analysis

Demographic data were compared between women and men using chi-squared tests, independent *t* test, or Mann-Whitney *U* tests where appropriate. All variables were checked for outliers and normality of residuals using Q-Q plots. TMT-data and CSF (p) Tau levels were log-transformed to meet assumptions of normality. Other outcome variables met the assumption of normally distributed residuals. To evaluate sex differences in clinical outcome measures, we used general linear models (GLM) with sex as the factor and baseline cognitive data or core clinical features as main outcome measures. Analyses were adjusted for age (clinical data) or age and education (cognitive data). Next, we performed general linear models (GLM) with sex as the factor and CSF biomarkers (i.e., α-syn Aβ1-42, Tau, and pTau) as the main outcome measure for each of the four CSF biomarkers separately. Analyses were adjusted for age (model 1) or age and APOEe4 genotype (model 2). A *p* < 0.05 was considered significant. Analyses were conducted using R statistical program (version 3.5.2 “eggshell igloo”).

## Results

Baseline characteristics are shown in Table 1. The majority of our sample consisted of men (f 17% vs m 83%). Women were older than men (f 70 vs m 68; *p* = 0.04), exhibited a shorter duration of complaints before diagnosis (f 2.3 vs m 3.5, *p* < 0.001), and more often reported visual hallucinations (f 58% vs m 38%, *p* < 0.02). Additionally, women more frequently carried the APOEe4 allele (f 70% vs m 52%, *p* = 0.08). No sex differences were found in other core clinical features, depressive symptoms, or years of education. After correcting for age and education, MMSE scores and animal fluency scores were lower in women than men. Performance on RAVLT (immediate and delayed recall), VAT, TMT-A, and TMT-B/A did not differ between sexes (Table 1).

**Table 1:** Baseline characteristics

	Women, *n* = 39	Men, *n* = 184	***p*** value
**Age**	70.1 ± 6.0	67.7 ± 7.3	**0.04**
**Education, years**	11.0 ± 2.8	11.4 ± 2.9	0.42
**Duration of complaints, years**	2.3 ± 1.2	3.5 ± 2.8	**< 0.001**
**APOE ɛ4 carrier,*n*(%)**	22 (70%)	86 (52%)	0.08
**Geriatric Depression Scale (0–15)**	4 ± 3	4 ± 3	0.65
**Core clinical features,*n*(%)**			
Visual hallucinations, *n* = 222	23 (59%)	69 (38%)	**0.02**
Parkinsonism, *n* = 216	23 (62%)	130 (73%)	0.28
RBD, *n* = 156	11 (55%)	99 (73%)	0.17
Cognitive fluctuations, *n* = 173	29 (94%)	119 (84%)	0.26
**Cognitive test scores**^**a**^**,*n*** **= 205**			
MMSE	21 ± 6	23 ± 4	**0.02**
RAVLT—immediate recall	− 1.94 ± 1.10	− 2.00 ± 0.95	0.74
RAVLT—delayed recall	− 1.80 ± 0.98	− 1.72 ± 0.96	0.84
VAT—12 items	− 6.20 ± 5.78	− 4.87 ± 4.99	0.31
TMT-A^b^	− 5.47 ± 6.46	− 4.60 ± 4.84	0.52
TMT-B/A^b,c^	− 6.93 ± 7.03	− 5.33 ± 6.42	0.32
Animal fluency	− 2.38 ± 1.02	− 1.78 ± 0.99	**0.002**
**Biomarker status, abnormal/available,*n*(%)**			
DAT SPECT, *n* = 107	15/15 (100%)	79/91 (87%)	**0.06**
CSF Aβ42 status	30/39 (77%)	104/186 (56%)	**0.02**
Abnormal Tau/Aβ42 ratio (> 0.52)	21/38 (55%)	55/185 (30%)	**0.002**

Data represent mean ± SD, median [IQR] or *n* (%). Parkinsonism: bradykinesia, rigidity, or tremor

*MMSE* Mini Mental State Examination, *RAVLT* Raven auditory verbal learning test (Dutch version), *VAT* Visual Association Test, *TMT* Trail Making Test

^a^Adjusted for age and education. Data of RAVLT, VAT, TMT, and animal fluency represent *z* scores, based on cognitively healthy subjects

^b^Data are inverted and log-transformed

^c^Missing values are imputed based on TMT-B/A ratio for *n* = 101

**Fig. 1:**
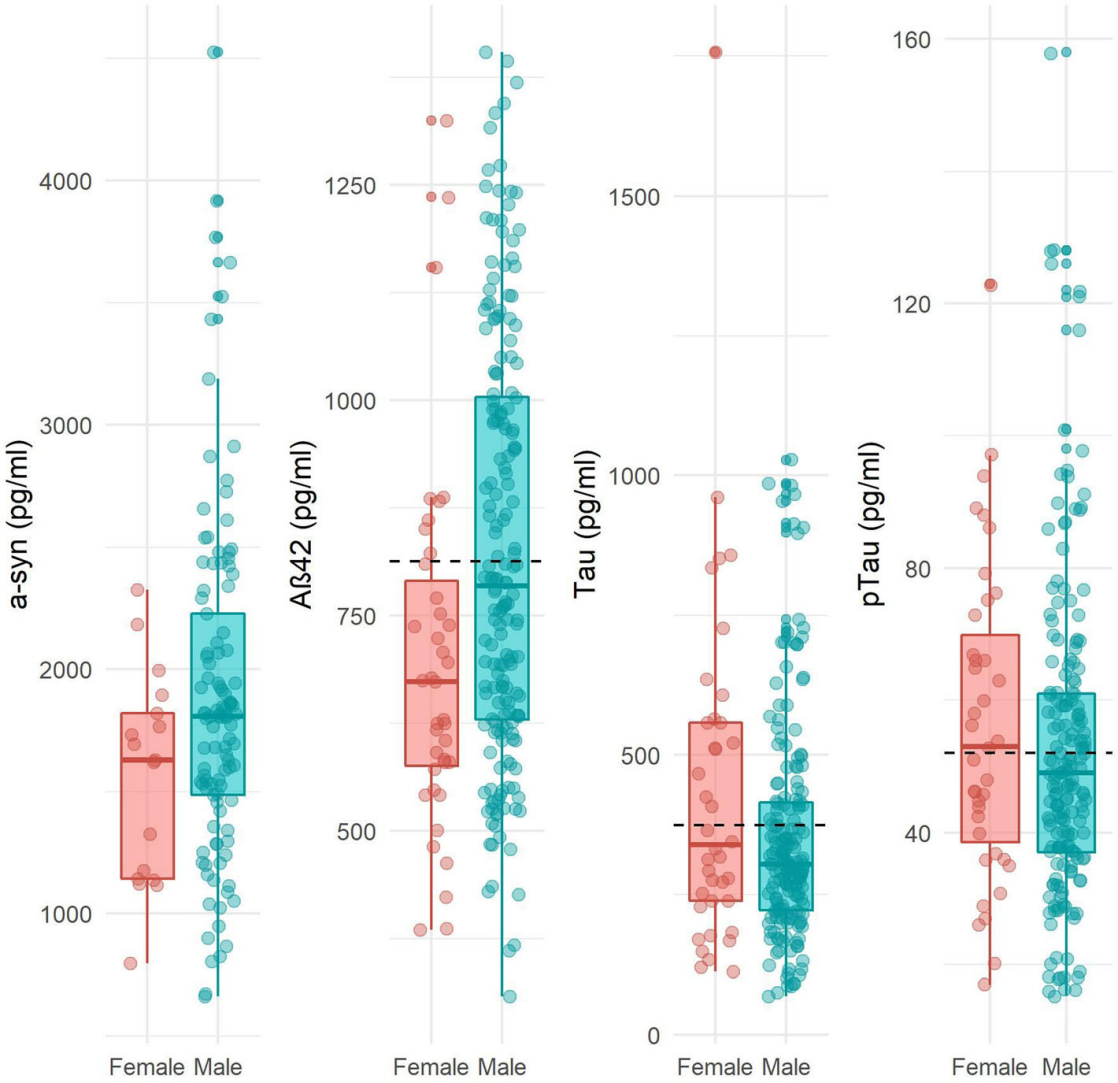
Sex differences in cerebrospinal fluid biomarker concentrations. Mean differences in CSF biomarker concentrations in picorgrams/milliliter between women and men. *p < 0.05. Abbreviations: CSF, cerebrospinal fluid; α-syn; alpha-synuclein; Aβ42; amyloid-β42; Tau, total tau; (p) Tau, tau phosphorylated at threonine 181. Dashed line reflects cutoffs for Aβ42 (813 pg/ml), Tau (375 pg/ml), and (p) Tau (52 pg/ml)

Next, we evaluated sex differences in CSF biomarker concentrations (Table 2; Fig. 1). Adjusted for age, women exhibited lower α-syn concentrations compared to men. Moreover, women had lower Aβ42 concentrations than men and exhibited more often abnormal CSF amyloid concentrations (f 77% vs m 56%, Table 1). No sex differences were found for CSF Tau or pTau concentrations. After adjusting for age and APOEe4 genotype, results for all CSF biomarkers remained largely similar (Table 2). When repeating the analyses in the subgroup with positive DAT-SPECT scans (*n* = 92; f 15 (16%), m 79 (84%)), we found similar effect sizes for all CSF biomarkers, although significance for α-syn was lost due to the loss of power (eTable 1).

**Table 2:** Cerebrospinal fluid concentrations at baseline

	Women, *n* = 39	Men, *n* = 184	Model 1^**a**^		Model 2^**b**^	
			Adjusted mean difference β coefficient (95%CI)	***p*** value	Adjusted mean difference β coefficient (95%CI)	***p*** value
**α-syn**^**c**^	1558 ± 431	1895 ± 707	− 390 (− 731:− 48)	**0.03**	**− 309 (− 657:− 40)**	**0.08**
**Aβ42**	696 ± 210	824 ± 251	− 108 (− 190:− 26)	**0.01**	**−103 (− 191:− 14)**	**0.02**
**Tau**^**d**^	5.9 ± 0.6	5.7 ± 0.5	0.10 (− 0.09:− 0.29)	0.31	0.06 (− 0.14:− 0.03)	0.55
**pTau**^**d**^	3.9 ± 0.5	3.9 ± 0.5	0.03 (− 0.13:0.19)	0.74	0.01 (−0.15:0.17)	0.90

Data presented as mean ± SD

^a^Model 1: adjusted for age

^b^Model 2: adjusted for age and APOEe4 genotype

^c^α-syn results were available for *n* = 117 (*f* = 17; *m* = 100)

^d^Analyses were performed with LN transformed data to meet assumptions of normality

## Discussion

In the present study, where DLB was more prevalent in men than in women, women had lower CSF Aβ42 and α-syn concentrations. In addition, women had more frequent hallucinations, had shorter duration of complaints before diagnosis, were older, and had lower scores on MMSE and fluency-tasks. Based on our findings, one could hypothesize that women have a more aggressive disease course compared to men.

When using predefined cutoffs for CSF Aβ42, 77% of women had amyloid pathology, in contrast to 56% of men. This suggests that women more often had concomitant AD pathology, whereas men more often had pure DLB pathology. These in vivo findings are in line with previous autopsy studies that observed that men were more likely to die with pure DLB pathology and women more often died with DLB + AD pathology [11, 30]. However, AD pathology is not only characterized by the presence of amyloid plaques, but also by NFT aggregation, which results in higher (p) Tau levels in CSF. As women in our study showed isolated lowered CSF Aβ42 levels, an alternative explanation could be that the lower levels of Aβ42 are directly related to the DLB disease process, rather than reflecting concomitant AD pathology. Previous studies have shown that Aβ42 induces the formation of high-molecular-weight α -syn, and vice versa [31]. Therefore, this synergistic interaction between Aβ42 and α -syn could lead to increased aggregation and accumulation of both α-syn and amyloid pathology in the women in our study compared to men [31–33].

Concomitant amyloid pathology in DLB has previously been related to a more severe clinical disease burden [12]. In line with these former findings, women in our cohort had lower MMSE and fluency test scores, and more frequent hallucinations, but a shorter duration of symptoms before diagnosis, suggesting a more aggressive disease course upon diagnosis in women with DLB. This could well be related to the amyloid-pathology that is more often encountered in DLB women. In our study, duration of complaints was estimated retrospectively; therefore, we cannot completely rule out that women with DLB were already in a later disease stage at the moment of diagnosis. Consistent with our hypothesis of a more aggressive disease course in women, one other study found that women with DLB have shorter survival after onset of dementia [34]. Prospective studies with longitudinal data are needed to further investigate sex differences in disease course over time.

Our data are in line with former observations of a strong male-predominance in DLB, as 83% of patients in our cohort were men [4–7]. The strong male-predominance in DLB could be explained by hormonal differences between sexes. Several previous studies have shown that 17β-estradiol (E2) conveys neuroprotective effects in women [35]. In fact, a previous in vitro study has shown that estradiol exhibits dose-dependent inhibition of α-syn aggregation and destabilization of preformed α-syn, which is thought to be driven by the antioxidant properties of estrogens [36–39]. This suggests that until menopause occurs in women, at the average age of 55 years, they are protected against the development of diseases such as DLB and Parkinson’s disease. During the second half of menopause, however, women show a sudden drop of estradiol levels [40], thus triggering the aggregation of α-syn and impeding the breakdown of preformed α-syn. If this is the case, sex differences in prevalence are expected to be more balanced with increasing age. In line with this hypothesis, a recent study reported a higher DLB prevalence of men than women under the age of 75 years, which equalized between the ages of 75 and 80 years. Above the age of 80 years, however, DLB was more common in women [8]. Future studies will have to demonstrate whether this shift in prevalence is mediated by hormonal differences.

Another explanation for the higher prevalence of DLB in men could be that concomitant AD pathology seen in DLB women potentially introduces a bias towards diagnosing these women with AD rather than DLB, therefore underdiagnosing the true proportion of women with DLB. In line with this reasoning, one study found that concomitant AD pathology decreased diagnostic accuracy, as typical AD symptoms were more pronounced, in contrast to DLB symptoms [41]. Our results imply that clinicians should be conscious of the risk of underdiagnosing women with DLB.

Our study has several strengths, including our well-defined, large sample of DLB patients. The clinical diagnosis of DLB was largely supported by DAT-SPECT imaging. In addition, we had CSF α-syn available in a considerable part of patients and CSF AD biomarkers in all patients. Furthermore, CSF collection follows a highly standardized protocol, assuring that the possibility of pre-analytical confounders is minimized [19]. Among the limitations is the retrospective design of our study. Information on RBD and fluctuations was rated retrospectively based on patients’ medical charts. In some cases, these features were not reported, which could either mean that they were not present or that it was not asked by the medical doctor. Therefore, there was a relatively large number of missing values for these features and there is a risk of underreporting. Future prospective studies should use standardized methods to assess these symptoms. A second limitation is that CSF total α-syn is not yet validated as a clinically useful marker in DLB, and there might be differences in sensitivity between different α-syn species. For example, soluble α-syn oligomers could be more useful in DLB, because oligomeric forms of α-syn seem to play a more essential role in the pathogenesis of α-synucleinopathies than t-α-syn [42]. Another α-syn species of interest is α-syn phosphorylated at serine 129 (pSer129-α-syn), as approximately 90% of accumulated α-syn in Lewy bodies consists of pSer129-α-syn [43]. However, these species cannot yet be measured robustly with commercial assays as these have not yet been validated. A last limitation was that a small part of our patients had a normal DAT-SPECT scan, not supportive of DLB diagnosis. However, clinical diagnosis was made carefully in a tertiary memory clinic setting and false-negative DAT-SPECT scans in early disease stages have been previously described [44].

## Conclusions

To conclude, our results indicate sex differences in DLB, wherein concomitant AD pathology was more frequent in women and CSF alpha-synuclein levels were lower in women compared to men. Concomitant pathology, combined with lower cognitive test scores and a shorter duration of complaints before diagnosis, could suggest a higher disease severity in women. Our results raise awareness among clinicians of the possibility that women are underdiagnosed and have the potential to aid in defining sex-specific personalized medicine strategies within DLB.

## Supplementary information

**Additional file 1: eTable 1.** Cerebrospinal fluid concentrations at baseline in DAT SPECT positive subgroup.

## Data Availability

All data used and/or analyzed during the present study are available from the corresponding author on reasonable request.

## Abbreviations

DLB: Dementia with Lewy bodies
CSF: Cerebrospinal fluid
Aβ: Amyloid-β
Aβ42: Amyloid β1-42
α-syn: Alpha-synuclein
Tau: Total tau
pTau: Tau phosphorylated at threonine 181
CV: Coefficient of variation
MMSE: Mini Mental State Examination
GLM: General linear models
RBD: REM sleep behavior disorder
NFT: Neurofibrillary tangles
AD: Alzheimer’s disease
MRI: Magnetic resonance imaging
EEG: Electroencephalography
DAT-SPECT: FP-CIT single-photon emission computed tomography
NPI: Neuropsychiatric inventory
TMT: Trail Making Test
RAVLT: Verbal learning test
Hb: Hemoglobuline
