# Supplementary material for: Sex-specific associations with cerebrospinal fluid biomarkers in dementia with Lewy bodies
Source: Alzheimers Res Ther. 2020 Apr 17;12:44. doi: 10.1186/s13195-020-00610-9 (PMC7165383; doi:10.1186/s13195-020-00610-9)
Supplement: Supplementary file 1 — Additional file 1: eTable 1. Cerebrospinal fluid concentrations at baseline in DAT SPECT positive subgroup. [file 13195_2020_610_MOESM1_ESM.docx]

**Supplementary table**

**eTable 1. Cerebrospinal fluid concentrations at baseline in DAT SPECT positive subgroup**

|  | **Women**  *n=15* | **Male**  *n= 77* | **Adjusted mean difference β coefficient (95%CI)^a^** | ***p*-value** |
| --- | --- | --- | --- | --- |
| **α-syn ^b^** | 1457±471 | 1767 ±595 | -349.5 (-898.4: 199.4) | 0.21 |
| **Aβ42** | 684 ±282 | 825 ±244 | -137.1 (-277.7: 2.6) | **0.05** |
| **Tau ^c^** | 5.9 ±0.8 | 5.8 ±0.5 | 0.1 (-0.2: 0.4) | 0.47 |
| **pTau ^c^** | 3.8 ±0.5 | 3.9 ±0.4 | -0.1 (-0.4: 0.2) | 0.72 |

Data presented as mean ± SD.

1. Adjusted for age
2. α-syn analysis was performed in a subset of patients *n=45 (f=5 ; m=40 )*
3. Age adjusted analyses were performed with LN transformed data to meet assuptions of normality.
